# Supplementary material for: The causal role of circulating inflammatory markers in osteoporosis: a bidirectional Mendelian randomized study
Source: Front Immunol. 2024 Jul 18;15:1412298. doi: 10.3389/fimmu.2024.1412298 (PMC11291241; doi:10.3389/fimmu.2024.1412298)
Supplement: Supplementary file 1 [file DataSheet_1.zip › supplementary material/OSTEOPOROSIS-CIM/GCST90274811.tsv.gz_forest.pdf]

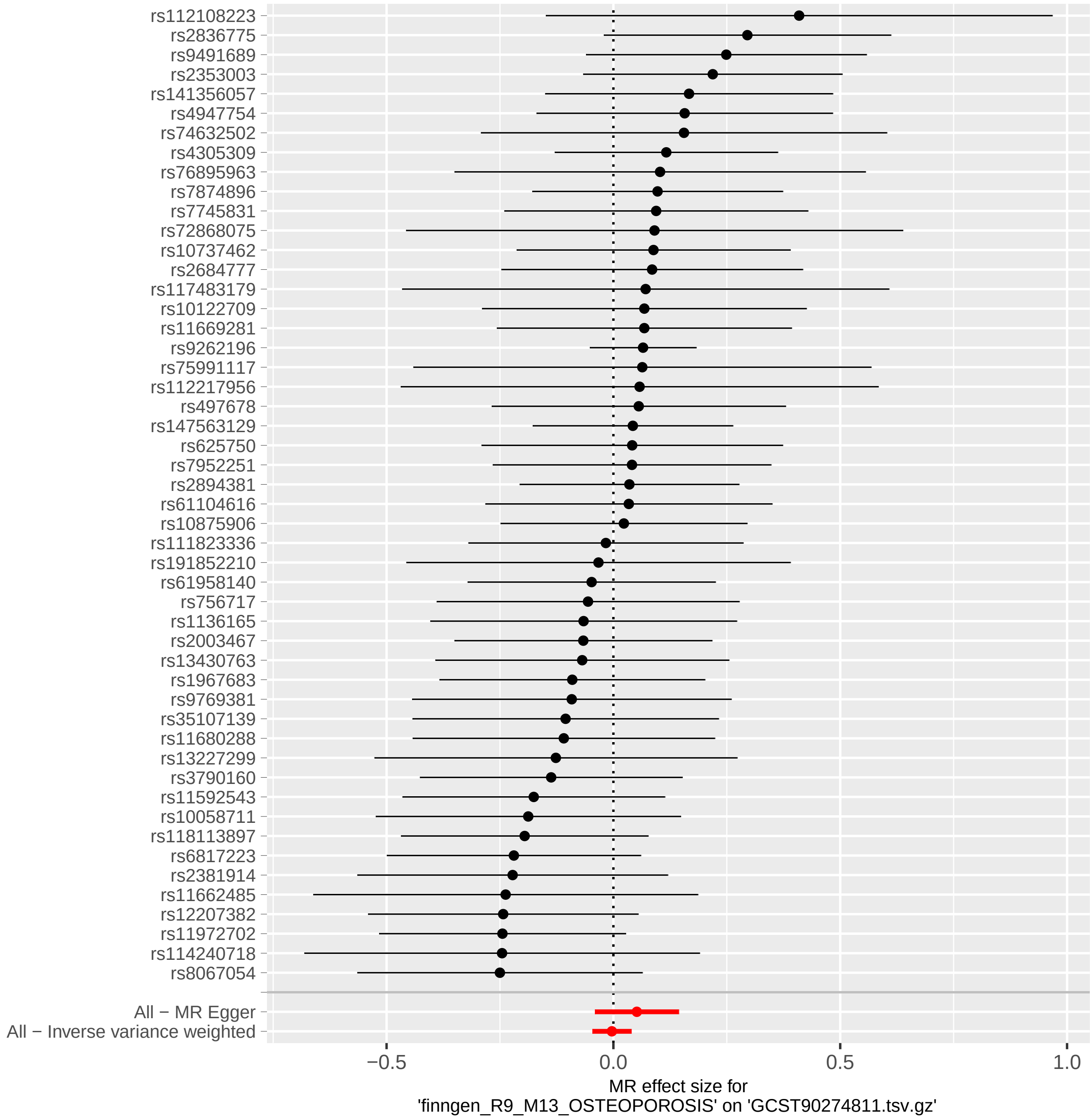
rs112108223
rs2836775
rs9491689
rs2353003
rs141356057
rs4947754
rs74632502
rs4305309
rs76895963
rs7874896
rs7745831
rs72868075
rs10737462
rs2684777
rs117483179
rs10122709
rs11669281
rs9262196
rs75991117
rs112217956
rs497678
rs147563129
rs625750
rs7952251
rs2894381
rs61104616
rs10875906
rs111823336
rs191852210
rs61958140
rs756717
rs1136165
rs2003467
rs13430763
rs1967683
rs9769381
rs35107139
rs11680288
rs13227299
rs3790160
rs11592543
rs10058711
rs118113897
rs6817223
rs2381914
rs11662485
rs12207382
rs11972702
rs114240718
rs8067054
All − MR Egger
All − Inverse variance weighted
−0.5
0.0
0.5
1.0
MR effect size for
'finngen_R9_M13_OSTEOPOROSIS' on 'GCST90274811.tsv.gz'
